# Psychometric Properties of the Brazilian Version of the Eating Attitudes Test in University Students

**DOI:** 10.1590/0034-7167-2024-0582

**Published:** 2025-12-19

**Authors:** Manuela de Mendonça Figueirêdo Coelho, Tifanny Horta Castro, Beatriz Alves de Oliveira, Jamylle Lucas Diniz, Mariana Cavalcante Martins, Janaína Fonseca Victor Coutinho, Fabiane do Amaral Gubert, Mônica Oliveira Batista Oriá

**Affiliations:** I Universidade Federal do Ceará. Fortaleza, Ceará, Brazil

**Keywords:** Feeding and Eating Disorders, Validation Study, Psychometrics, Students, Attitude, Trastornos de Alimentación y de la Ingestión de Alimentos, Estudio de Validación, Psicometría, Estudiantes, Actitud

## Abstract

**Objectives:**

to evaluate the psychometric properties of the Brazilian version of the Eating Attitudes Test among university students and to analyze potential differences between sexes.

**Methods:**

a methodological, quantitative study was conducted with 5,292 students. Data were collected through an online questionnaire comprising 26 Likert-scale items, including sociodemographic, academic, and health-related questions. The psychometric properties assessed included factorial validity, internal consistency, psychometric sensitivity, and structural equivalence between groups.

**Results:**

confirmatory factor analysis supported the multidimensional structure of the instrument, with satisfactory fit indices. The chi-square statistic was 14,447.944 with 296 degrees of freedom (p < 0.001), chi-square/df ratio of 48.81, comparative fit index of 0.843, Tucker-Lewis index of 0.828, root mean square error of approximation of 0.095, and standardized root mean square residual of 0.125. Structural equivalence was observed between sexes.

**Conclusions:**

the instrument demonstrated robust factorial validity for use among Brazilian university students.

## RESUMEN

**Objetivos:**

evaluar las propiedades psicométricas de la versión brasileña del Test de Actitudes Alimentarias en estudiantes universitarios y analizar posibles diferencias entre los sexos.

**Métodos:**

estudio metodológico con enfoque cuantitativo, realizado con 5.292 estudiantes. La recolección de datos se realizó mediante un cuestionario en línea con 26 ítems en escala de tipo Likert, incluyendo información sociodemográfica, académica y de salud. Se evaluaron la validez factorial, la consistencia interna, la sensibilidad psicométrica y la equivalencia estructural entre grupos.

**Resultados:**

el análisis factorial confirmatorio respaldó la estructura multidimensional del instrumento, con índices de ajuste satisfactorios. El valor de chi-cuadrado fue de 14.447,944 con 296 grados de libertad (p < 0,001), la razón chi-cuadrado/grados de libertad fue de 48,81, el índice comparativo de ajuste fue de 0,843, el índice de Tucker-Lewis de 0,828, el error cuadrático medio de aproximación de 0,095 y el residuo cuadrático medio estandarizado de 0,125. Se observó equivalencia estructural entre los sexos.

**Conclusiones:**

el instrumento demostró una validez factorial robusta para su uso en estudiantes universitarios brasileños.

## INTRODUCTION

Eating disorders (EDs) are serious conditions resulting from disruptions in eating and psychological behaviors, with significant repercussions in physical, psychological, social, and economic domains^(1)^. Among the main EDs, anorexia nervosa, bulimia nervosa, and binge eating disorder (BED) are the most prominent^(1)^. Although these conditions can affect individuals of all ages and genders, they are more commonly observed during adolescence and young adulthood, particularly among women^(2)^. The prevalence of EDs is 5.7% in women and 2.2% in men^(3)^.

University students, in particular, are at high risk of developing EDs^(4)^. Factors such as academic stress, changes in daily routines, and social adaptation can trigger the onset of EDs^(5)^. A meta-analysis indicated that 19.7% of university students are at risk of developing EDs^(6)^. Researchers identified a significant increase in EDs among male and female university students between 2009 and 2021^(4)^. Notably, the frequency of EDs among females is 2.5 times higher (OR: 2.55, 95% CI: 1.22–5.32, p = 0.012) compared to males^(5)^.

The Eating Attitudes Test (EAT), developed by Garner and Garfinkel (1979), aims to screen for symptoms and risk behaviors associated with the development of EDs. Initially composed of 40 multiple-choice items, the EAT was later shortened to 26 items (EAT-26), with a cutoff score of 21. The items are organized into three factors: 1) Dieting, which addresses the refusal of high-calorie foods and excessive concern with body image; 2) Bulimia and Food Preoccupation, related to binge eating episodes followed by compensatory behaviors to prevent weight gain; 3) Oral Control, which assesses self-control over eating and the influence of the environment on food intake. A score above 21 indicates a positive screening, suggesting the need for further clinical evaluation. This instrument was validated by the authors in a Canadian female population. Although initially composed of 40 multiple-choice items, a shortened version with 26 items (EAT-26) was later proposed, with a cutoff of 21 points^(7)^.

Due to its ease of application, the EAT has been translated and validated in several countries^(8-12)^. Studies in Malaysia, for example, suggested that the hypothetical three-factor model of the EAT-26 did not show a satisfactory fit for undergraduate student data^(13)^. In Spain, analysis revealed robust internal consistency with six factors, while in Colombia, researchers identified a four-factor structure^(14)^. The study by Ocker et al. indicated that the three-factor model of the EAT-26 demonstrated poor model fit indices^(15)^. In Portugal, internal consistency and test-retest reliability were satisfactory, resulting in four factors in the total sample^(16)^. In Brazil, one study with women showed acceptable internal consistency, while another with men found that 32.8% of the data variance was explained by a single factor, confirming its internal consistency (α > 0.88^)(17,18)^.

The Brazilian scientific literature reveals a lack of studies exploring the factorial validity of the EAT-26 in samples of university students of both sexes. Most research focuses on specific populations, such as male or female adolescents^(8,19)^. However, an exhaustive literature search revealed a significant gap regarding the factorial validation of the EAT-26 in mixed university student populations, highlighting the need for future studies that consider both sexes in this context.

For nursing practice, the relevance of the study lies in the potential use of the EAT-26 as an early screening tool, enabling nurses to identify students at risk for EDs and implement prevention and intervention strategies. Moreover, the validation of the test supports its use in mental health and nutrition promotion programs within academic settings, strengthening the role of nursing in the interdisciplinary management of EDs.

Therefore, this study aims to evaluate the psychometric properties of the Brazilian version of the EAT among university students, analyzing potential differences between sexes. It is hypothesized that the Brazilian version of the EAT-26 presents good psychometric properties and that significant differences exist between sexes in eating attitudes, with one group at greater risk of disordered eating behaviors.

## OBJECTIVES

To evaluate the psychometric properties of the Brazilian version of the Eating Attitudes Test among university students, analyzing potential differences between sexes.

## METHODS

### Ethical Aspects

All ethical standards were observed, and the study was approved by the Research Ethics Committee of the Federal University of Ceará, under opinion no. 4.277.440.

### Design

A methodological psychometric validation study with a quantitative approach was conducted, focusing on the structural validation and reliability of the EAT-26 in the Brazilian university population. The study followed the guidelines of the COSMIN Reporting Guideline, which served as the guiding framework for its organization and writing.

### Population and Sample

The sample consisted of Brazilian university students from both public and private institutions. The inclusion criteria were being over 18 years of age and having completed at least the first semester of undergraduate studies. Incomplete questionnaires were excluded, resulting in a final sample of 5,292 participants out of a total of 5,345 respondents.

Data collection was carried out using an online questionnaire (Google Forms), which included sociodemographic, academic, and health information, as well as the EAT-26, composed of 26 Likert-scale items distributed across the three previously mentioned factors. To select the participating higher education institutions, state and federal universities were identified through the Brazilian Ministry of Education (MEC in Portuguese) website. Then, the websites of all institutions were located via Google, from which the email lists of course coordinators were gathered. The research materials were sent via email to the coordinators, requesting that they disseminate the questionnaire to enrolled students. According to the MEC census, Brazil has approximately 9 million university students, with only 23.2% (2,079,123) enrolled in public institutions^(20)^. To determine the sample size, a 95% confidence level and a 3% margin of error were considered, resulting in a minimum required sample of 1,066 students. The calculation was performed using an online sample size calculator. Data collection occurred between November 2021 and March 2022.

The participants were enrolled in programs in Health Sciences, Applied Social Sciences, Linguistics, Language and Arts, Engineering, Biological Sciences, and Agricultural Sciences, ensuring representation from all academic areas and from all Brazilian states and federal units.

### Data Analysis

The psychometric sensitivity of the EAT items was assessed using measures of central tendency, dispersion, kurtosis, and skewness, with items showing skewness coefficients < 3 and kurtosis < 7 considered acceptable^(21)^. Internal consistency was estimated using Cronbach’s alpha coefficient (α), with α ≥ 0.70 considered adequate. Sample adequacy was verified using the Kaiser-Meyer-Olkin (KMO) index, with values above 0.5 considered acceptable, and Bartlett’s test of sphericity (p < 0.005).

To assess the correlation between factors, Spearman’s correlation test was used, with the following interpretation: 0.8 to 1.0 as strong correlation; 0.5 to 0.8 as moderate; 0.2 to 0.5 as weak; and 0.0 to 0.2 as insignificant.

Confirmatory factor analysis (CFA) was performed to evaluate the plausibility of the EAT’s multidimensional structure. CFA is a technique used, among various purposes, to determine whether the factor structure of a proposed model aligns with the theoretical concept of the construct in a specific sample. As a confirmatory test, the factor structure must be defined by the researcher prior to conducting the analysis^(22)^.

The analysis was conducted using the Robust Diagonally Weighted Least Squares (RDWLS) estimation method, along with a configural model assessment to verify the equivalence of item categories between males and females^(23,24)^. The fit indices used included: chi-square (χ²), chi-square to degrees of freedom ratio (χ²/df), Comparative Fit Index (CFI), Tucker-Lewis Index (TLI), Standardized Root Mean Residual (SRMR), and Root Mean Square Error of Approximation (RMSEA). χ² values should not be significant; χ²/df should be < 5, preferably < 3; CFI and TLI should be > 0.90, preferably above 0.95; RMSEA should be < 0.08, preferably < 0.06, with a confidence interval (upper bound) < 0.10^(25)^.

Factor loadings above 0.40 were considered adequate^(26)^. In addition to administering the scale, sociodemographic characteristics were collected and analyzed, including age, sex, marital status, state of residence, family income, and academic major.

The results were organized in an Excel spreadsheet and exported to the Statistical Package for the Social Sciences (SPSS), version 23.0, for descriptive, inferential, and correlational analyses. CFA was conducted using Jeffreys’s Amazing Statistics Program (JASP).

## RESULTS

The sample consisted of 5,292 university students from all regions of Brazil, with a mean age of 24 years (SD ± 6.9). Regarding sex, 67.4% (n = 3,565) were women; 45.5% (n = 2,408) self-identified as white; 86.5% (n = 4,577) were single; and 49.5% (n = 2,619) reported a family income between 1 and 5 minimum wages. Participants represented all regions of the country, with the highest proportion enrolled in Humanities programs (20.8%, n = 1,101) and in federal institutions (57.9%, n = 3,066).

Correlations among the scale factors were analyzed. Although the factors showed statistically significant correlations (p = 0.000), a moderate correlation was observed between the Dieting factor and the Bulimia and Food Preoccupation factor (rho = 0.637), while the Oral Control factor showed weak correlations with the other factors (rho = 0.284 and rho = 0.209). Descriptive data indicated the psychometric sensitivity of the EAT items (Table 1). Kurtosis values were not significant, ranging from -1.456 to 6.780.

**Table 1:** Summary and distribution measures by item of the Eating Attitudes Test for Brazilian university students (N = 5,292), Fortaleza, Ceará, Brazil, 2021–2022

Item	Mean	Standard Deviation	Skewness	Kurtosis
I1) I am terrified about gaining weight	1.01	1.242	0.662	-1.277
I2) I avoid eating when I am hungry	0.28	0.741	2.685	6.046
I3) I am preoccupied with food	0.72	1.050	1.172	-0.081
I4) Overeating makes me feel like I can’t stop	0.66	1.093	1.310	0.075
I5) I cut my food into small pieces	0.48	0.928	1.820	1.922
I6) I pay attention to the number of calories in the food I eat	0.35	0.846	2.318	3.965
I7) I particularly avoid foods with high carbohydrate content (bread, potatoes, rice)	0.28	0.752	2.705	6.138
I8) I feel that others would like me to eat more	0.54	1.038	1.622	0.972
I9) I vomit after eating	0.14	0.569	2.239	6.000
I10) I feel extremely guilty after eating	0.56	1.044	1.561	0.791
I11) I am concerned about the desire to be thinner	0.89	1.228	0.877	-0.984
I12) I think about burning off calories when I exercise	0.89	1.209	0.857	-0.977
I13) People think that I am too thin	0.56	1.036	1.565	0.818
I14) I am concerned about the idea of fat on my body	0.86	1.202	0.907	-0.892
I15) I take longer to eat meals than other people	0.69	1.117	1.260	-0.082
I16) I avoid foods that contain sugar	0.34	0.806	2.369	4.378
I17) I usually eat diet foods	0.27	0.739	2.798	6.652
I18) I feel that food controls my life	0.44	0.946	1.940	1.203
I19) I show self-control around food	0.87	1.105	0.851	-0.785
I20) I feel pressured by others to eat	0.43	0.923	1.997	2.477
I21) I spend a lot of time thinking about food	0.62	1.033	1.405	0.443
I22) I feel uncomfortable after eating sweets	0.45	0.948	1.891	2.032
I23) I go on diets to lose weight	0.44	0.933	1.940	2.238
I24) I enjoy feeling my stomach empty	0.31	0.816	2.566	5.113
I25) I enjoy trying new high-calorie foods	1.28	1.187	0.268	-1.456
I26) I feel like vomiting after meals	0.24	0.729	3.018	6.780

*N – number of participants.*

Cronbach’s alpha for the Dieting factor was α = 0.827; for Bulimia, α = 0.770; and for Oral Control, α = 0.725. The internal consistency of the EAT was adequate (α = 0.889), with a KMO index of 0.921 and a significant Bartlett’s test of sphericity (p < 0.001).

CFA was conducted to assess the plausibility of the EAT’s multidimensional structure(28). The model showed good fit indices. The results were χ² = 14,447.944 (296 df), p < 0.001. The chi-square to degrees of freedom ratio was high (48.81), with CFI = 0.843, TLI = 0.828, SRMR = 0.125, and RMSEA = 0.095 [0.094–0.096], which is consistent with previously reported unsatisfactory fit criteria for the factorial structure in this sample. The item factor loadings were high, ranging from 0.43 (item I7) to 1.31 (item I16) (Model 01).

Item I10 – I feel extremely guilty after eating (λ = 0.980) showed high cross-loadings with I8 – I feel that others would like me to eat more (λ = 0.886), I13 – People think that I am too thin (λ = 0.931), and I20 – I feel pressured by others to eat (λ = 0.634).

To identify the best fit, alternative factorial structures were tested to refine the original model by removing items with lower or problematic cross-loadings. Model 02 was tested by removing I20; Model 03 by removing items I20 and I8; and Model 04 by removing items I20, I8, and I13 (Table 2).

**Table 2:** Fit indices of the Confirmatory Factor Analysis of the Eating Attitudes Test applied to university students, Fortaleza, Ceará, Brazil, 2021–2022

Estimates	Model 01	Model 02	Model 03	Model 04
χ^2^	14447.944	12365.644	9974.759	6748.821
χ^2^/df	48.81	45.46	40.05	29.73
CFI	0.843	0.858	0.880	0.916
TLI	0.828	0.844	0.867	0.907
RMSEA	0.095 (0.094-0.096)	0.092 (0.090-0.093)	0.086 (0.084-0.087)	0.074 (0.072-0.075)
SRMR	0.125	0.120	0.114	0.097

*CFI – Comparative Fit Index; TLI – Tucker-Lewis Index; RMSEA – Root Mean Square Error of Approximation; SRMR – Standardized Root Mean Square Residual.*

All fit indices presented in Model 04 are acceptable for the original three-factor structure of the scale. Multigroup Confirmatory Factor Analysis (MG-CFA) was conducted to investigate whether the EAT demonstrates invariance between males and females. The analysis was performed for both the original model (Model 01) and Model 04, which showed the best fit for the tested sample (Table 3).

**Table 3:** Configural invariance indices from the Multigroup Confirmatory Factor Analysis (gender) of the Eating Attitudes Test applied to university students, Fortaleza, Ceará, Brazil, 2021–2022

Estimates	Model 01	Model 04
χ^2^	14443.225	6748.821
χ^2^/df	24.39	14.82
CFI	0.847	0.920
TLI	0.832	0.920
RMSEA	0.094 (0.093-0.095)	0.0742 (0.071-0.074)
SRMR	0.125	0.097

*CFI - Comparative Fit Index; TLI - Tucker Lewis Index; RMSEA - Root-Mean-Square Error of Aproximation; SRMR - Standardized root mean square residuals.*

**Figure 1:**
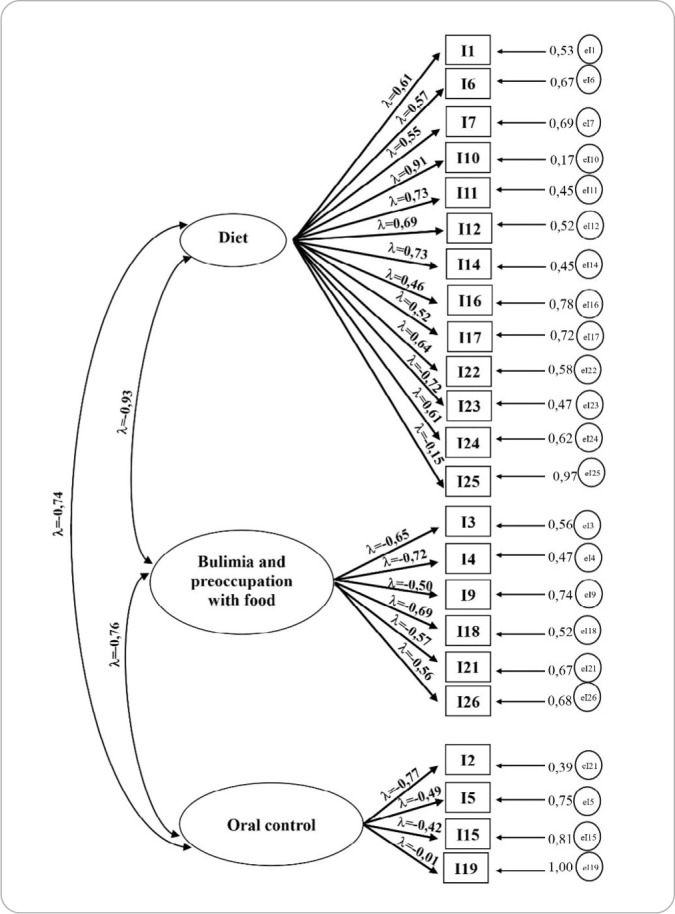
Factor analysis of the adjusted Eating Attitudes Test for a sample of Brazilian university students (n = 5,262)

**Figure 2:**
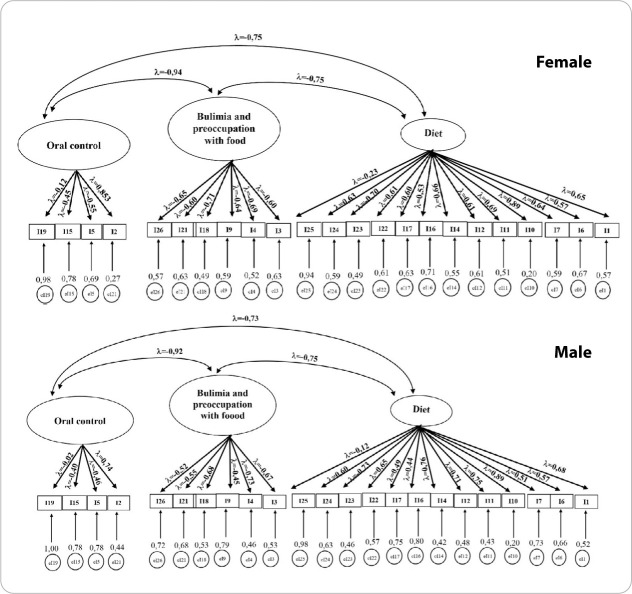
Factor analysis of the adjusted Eating Attitudes Test for a sample of Brazilian university students, female and male groups (n = 5,262)

Configural invariance demonstrated that Model 04 of the EAT functions as an equivalent measure for both males and females, allowing for group comparisons after refinement of the instrument. Figure 1 presents the factor loadings of the instrument after adjustment, and Figure 2 shows the factor loadings by group (male and female):

## DISCUSSION

The results of this study provide valuable insights into the factorial validity of the EAT-26 among Brazilian university students, significantly contributing to the literature on screening instruments for EDs in this specific population.

The EAT-26 is widely used to identify high-risk eating behaviors and is composed of three main factors: Dieting, Bulimia and Food Preoccupation, and Oral Control. The Dieting factor assesses the tendency to avoid high-calorie foods and engage in dietary restriction, reflecting behaviors associated with the pursuit of thinness and body dissatisfaction. Studies have indicated that high scores on this factor are correlated with restrictive practices and dissatisfaction with body image, especially among female adolescents^(27)^.

The second factor, Bulimia and Food Preoccupation, investigates binge eating episodes followed by compensatory behaviors, along with excessive preoccupation with food. High scores in this domain are associated with an increased risk of developing EDs such as bulimia nervosa and are frequently correlated with elevated levels of anxiety and impulsivity^(28)^.

Finally, the Oral Control factor relates to perceived self-control over food intake and social pressure to eat in a certain way. Individuals with low scores on this factor may show greater vulnerability to episodes of loss of control over eating, influenced by social and emotional contexts^(29)^.

CFA revealed that, after adjustments, the three-factor model of the EAT-26 showed acceptable fit indices for this sample, supporting its original structure. This finding differs from those reported in recent international studies. A study conducted with children in Spain showed that exploratory factor analysis supports a multidimensional structure of the scale, but with six factors^(29)^, indicating that the factorial structure of the EAT-26 may vary depending on cultural and age-related contexts.

The internal consistency of the instrument was adequate (α = 0.889), aligning with recent international studies. A meta-analysis^(6)^ reported an average reliability of 0.86 for the EAT-26 across various populations. The strong internal consistency reinforces the instrument’s reliability for use in Brazilian academic contexts, which is crucial given the increasing prevalence of high-risk eating behaviors among university students^(4,30)^.

A particularly noteworthy finding was the configural invariance demonstrated between men and women after the instrument was refined. This result is especially relevant in light of the gender differences often observed in EDs. It highlights the importance of considering gender differences in the manifestation and assessment of eating disorders, emphasizing the need for instruments that are sensitive to such variations. The configural invariance found in this study suggests that the adjusted EAT-26 may serve as an equivalent measure for both sexes, enabling valid group comparisons^(31)^.

Evidence of significant differences in the prevalence and manifestation of eating disorders between males and females has been documented in the literature. Although traditionally associated with women, these disorders have shown a notable increase among men, suggesting that sociocultural and biological factors influence both genders in distinct ways. Additionally, men often report feelings of shame for having a condition frequently perceived as “feminine”, which may lead to underreporting and delays in diagnosis^(8,32)^.

Sociocultural factors, such as pressure to maintain a specific body image, influence both genders but manifest in different ways. Thus, it is crucial that mental health professionals remain attentive to how these disorders present in different patients, considering gender-specific characteristics in the development and clinical presentation of eating disorders^(6,33)^.

The prevalence of high-risk behaviors related to eating disorders among university students highlights the importance of reliable and valid screening tools^(34)^. The EAT-26, with its factorial structure confirmed in this study, may serve as a valuable instrument for the early identification of students at risk^(35)^.

For the field of nursing, these findings have significant implications. Nurses working in university settings or primary care may now use the EAT-26 with greater confidence as a screening tool for risk behaviors associated with eating disorders. The validation of its factorial structure and the demonstration of gender invariance allow for broader and more equitable application of the instrument. This aligns with recommendations emphasizing the importance of evidence-based digital interventions for mental health among university students, in which validated screening tools play a crucial role^(35)^.

Moreover, confirming the validity of the EAT-26 in this specific population may guide the development of prevention and early intervention programs in mental health focused on eating disorders. Nurses can play a key role in implementing such programs, using the EAT-26 as an initial assessment tool and to monitor progress.

It is important to note that the study identified the need for adjustments to the original model, including the removal of some items to improve fit. This suggests that, while the EAT-26 is a valuable tool, there may be room for cultural or context-specific adaptations for the Brazilian university population. Future studies could explore the development of culturally adapted versions of the instrument, potentially enhancing its sensitivity and specificity in the Brazilian context.

### Study limitations

Regarding the limitations, it is noted that the study did not assess the concurrent or predictive validity of the instrument, which is an important aspect in the comprehensive evaluation of screening tools for eating disorders. The cross-sectional nature of the study does not allow for inferences about the temporal stability of the identified factorial structure.

Although the study provided robust evidence on the factorial validity of the EAT-26 among Brazilian university students, some limitations must be considered. Data collection through a self-reported online questionnaire may have introduced response bias, as participants may have underestimated or overestimated their eating behaviors due to factors such as subjective perception or social stigma. Although the sample was broad, it may not equally represent all regions of the country and university programs, which could affect the generalizability of the findings.

Another aspect to consider is the influence of sociocultural and psychological factors such as anxiety, depression, and body dissatisfaction, which were not thoroughly addressed but play a significant role in the development of eating disorders. Thus, future studies may benefit from the inclusion of complementary clinical evaluations, longitudinal analyses, and investigations into the impact of emotional and environmental factors on the manifestation of dysfunctional eating behaviors.

### Contributions to the field of nursing

The potential contributions of this study to the field of nursing lie in identifying a validated tool for screening risk behaviors related to eating disorders in university students, enabling early interventions. It also highlights the need for more targeted and effective prevention and health education programs based on local evidence.

Furthermore, the findings contribute to evidence-based nursing practice by providing data on the validity of a widely used instrument and paving the way for future research on the cultural adaptation of mental health instruments for the Brazilian population.

## CONCLUSIONS

In conclusion, this study provides robust evidence of the factorial validity of the EAT-26 among Brazilian university students, with important implications for mental health nursing and primary care practice. CFA indicated that, after adjustments, the EAT-26 model showed configural invariance, supporting its use as an equivalent measure for both men and women.

## Data Availability

The research data are available in a repository:https://osf.io/wz6u4/overview.
