# Supplementary material for: Psychometric Properties of the Brazilian Version of the Eating Attitudes Test in University Students
Source: Rev Bras Enferm. 2025 Dec 19;78(5):e20240582. doi: 10.1590/0034-7167-2024-0582 (PMC12716680; doi:10.1590/0034-7167-2024-0582)
Supplement: Translation

# Translation: Propriedades psicométricas da versão brasileira do Teste de Atitudes Alimentares em estudantes universitários

**DOI:** 10.1590/0034-7167-2024-0582pt

**Authors:** Manuela de Mendonça Figueirêdo Coelho, Tifanny Horta Castro, Beatriz Alves de Oliveira, Jamylle Lucas Diniz, Mariana Cavalcante Martins, Janaína Fonseca Victor Coutinho, Fabiane do Amaral Gubert, Mônica Oliveira Batista Oriá

**Affiliations:** I Universidade Federal do Ceará. Fortaleza, Ceará, Brasil

**Keywords:** Transtornos da Alimentação e da Ingestão de Alimentos, Estudo de Validação, Psicometria, Estudantes, Atitude

## Abstract

**Objetivos:**

avaliar as propriedades psicométricas da versão brasileira do Teste de Atitudes Alimentares em estudantes universitários e analisar possíveis diferenças entre os sexos.

**Métodos:**

estudo metodológico com abordagem quantitativa, realizado com 5.292 estudantes. A coleta foi feita por questionário online com 26 itens em escala do tipo Likert, incluindo dados sociodemográficos, acadêmicos e de saúde. Foram avaliadas validade fatorial, consistência interna, sensibilidade psicométrica e equivalência entre grupos.

**Resultados:**

a análise fatorial confirmou a estrutura multidimensional do instrumento, com bons índices de ajuste. A estatística qui-quadrado foi de 14.447,944 com 296 graus de liberdade, p menor que 0,001, razão qui-quadrado por graus de liberdade de 48,81, índice de comparação ajustado de 0,843, índice de Tucker-Lewis de 0,828, erro médio quadrático de aproximação de 0,095 e índice de resíduos padronizados de 0,125. A equivalência estrutural foi demonstrada entre os sexos.

**Conclusões:**

o instrumento demonstrou validade robusta para uso em estudantes universitários brasileiros.

## INTRODUÇÃO

Os transtornos alimentares (TA) são transtornos graves, que resultam de distúrbios no comportamento alimentar e psicológico, com repercussões significativas nos âmbitos físico, psicológico, social e econômico^(1)^. Dentre os principais TA, destacam-se a anorexia nervosa, a bulimia nervosa e o transtorno da compulsão alimentar (TCA)^(1)^. Embora essas condições possam afetar indivíduos de todas as idades ou sexos, elas são mais frequentemente observadas durante a adolescência e jovens adultos, especialmente em mulheres^(2)^. A prevalência de TA é de 5,7% em mulheres e 2,2% em homens^(3)^.

Os estudantes universitários, em especial, apresentam alto risco para desenvolver transtorno alimentar^(4)^. Fatores como estresse acadêmico, mudanças na rotina diária e adaptações sociais podem ocasionar o transtorno^(5)^. Estudo de meta-análise apontou que 19,7% dos universitários possuem risco de desenvolver TA^(6)^. Pesquisadores identificaram um aumento significativo de TA entre estudantes universitários dos sexos masculino e feminino entre 2009 e 2021^(4)^. Destaca-se que a frequência de TA em pessoas do sexo feminino é 2,5 vezes maior (OR: 2,55, IC 95%: 1,22–5,32, p = 0,012) em comparação aos homens^(5)^.

O Teste de Atitudes Alimentares (*Eating Attitudes Test* - EAT), criado por Garner e Garfinkel (1979), visa rastrear sintomas e comportamentos de risco para o desenvolvimento de TAs. Inicialmente composto por 40 itens de múltipla escolha, o EAT foi posteriormente abreviado para 26 itens (EAT-26), com um ponto de corte de 21. Os itens estão organizados em três fatores: 1) Dieta, que aborda a recusa de alimentos calóricos e a preocupação excessiva com a imagem corporal; 2) Bulimia e Preocupação com os Alimentos, relacionada a episódios de ingestão compulsiva seguidos de comportamentos para evitar ganho de peso; 3) Controle Oral, que analisa o autocontrole alimentar e a influência ambiental na ingestão. Uma pontuação superior a 21 indica um teste positivo, recomendando uma avaliação clínica adicional. Esse instrumento foi validado pelos autores com pessoas do sexo feminino no Canadá. Inicialmente, o instrumento continha 40 itens de múltipla escolha, todavia, foi proposta uma versão abreviada com 26 itens (EAT-26), sendo o ponto de corte de 21 pontos^(7)^.

O EAT, por ser um instrumento de fácil aplicação, já foi traduzido e validado em diversos países^(8-12)^. Estudos na Malásia, por exemplo, sugeriram que o modelo hipotético de três fatores do EAT-26 não alcançou um ajuste satisfatório para dados de estudantes de graduação^(13)^. Na Espanha, a análise revelou consistência interna robusta de seis fatores, enquanto na Colômbia, pesquisadores encontraram uma estrutura de quatro fatores^(14)^. A pesquisa de Ocker et al. apontou que os índices de ajuste do EAT-26 de três fatores representaram um ajuste de modelo inaceitável^(15)^. Em Portugal, a consistência interna e a confiabilidade teste-reteste foram satisfatórias, resultando em quatro fatores na amostra total^(16)^. No Brasil, um estudo realizado com mulheres mostrou consistência interna aceitável, enquanto outro com homens encontrou que 32,8% da variância dos dados foi explicada por um único fator, confirmando sua consistência interna (α>0,88)^(17,18)^.

A literatura científica brasileira apresenta uma escassez de estudos que exploram a validade fatorial do EAT-26 em amostras de estudantes universitários de ambos os sexos. A maioria das pesquisas concentra-se em populações específicas, como adolescentes do sexo feminino ou masculino^(8,19)^. No entanto, mediante busca exaustiva na literatura, observou-se lacuna significativa referente à validação fatorial do EAT-26 em populações mistas de universitários, o que ressalta a necessidade de investigações futuras que considerem ambos os sexos nesse contexto.

Para a prática de enfermagem, a relevância do estudo se destaca na possibilidade de uso do EAT-26 como ferramenta de triagem precoce, permitindo que enfermeiros identifiquem estudantes em risco de desenvolver TA e implementem estratégias de prevenção e intervenção. Além disso, a validação do teste possibilita sua utilização em programas de promoção da saúde mental e nutricional no ambiente acadêmico, fortalecendo o papel da enfermagem na abordagem interdisciplinar dos TA.

Assim, este estudo objetiva avaliar propriedades psicométricas da Versão Brasileira do EAT-26 em estudantes universitários, analisando possíveis diferenças entre os sexos. Acredita-se que a versão Brasileira do Teste EAT-26 apresenta boas propriedades psicométricas, e há diferenças significativas entre os sexos nas atitudes alimentares, com maior risco de comportamentos alimentares disfuncionais em um dos grupos.

## OBJETIVOS

Avaliar as propriedades psicométricas da versão brasileira do Teste de Atitudes Alimentares em estudantes universitários e analisar possíveis diferenças entre os sexos.

## MÉTODOS

### Aspectos Éticos

Os aspectos éticos foram respeitados, e o estudo foi aprovado pelo Comitê de Ética em Pesquisa da Universidade Federal do Ceará, com parecer n. 4.277.440.

### Desenho

Realizou-se um estudo metodológico de validação psicométrica, com abordagem quantitativa, focado na validação estrutural e confiabilidade do EAT-26 na população universitária brasileira. Seguiu-se as diretrizes do *COSMIN Reporting Guideline*, como instrumento norteador da organização e escrita deste estudo.

### População e Amostra

A amostra foi composta por universitários brasileiros de instituições públicas e privadas. O critério de inclusão foi ter mais de 18 anos e ter completado o primeiro semestre da graduação. Foram excluídos formulários incompletos, resultando em uma amostra final de 5.292 participantes, a partir de um total de 5.345 respondentes.

A coleta de dados foi realizada por meio de um questionário online (Google Forms), abrangendo informações sociodemográficas, acadêmicas e de saúde, incluindo o EAT-26, composto por 26 questões em escala Likert, distribuídas nos três fatores mencionados anteriormente. Para selecionar as instituições de ensino superior participantes, foram identificadas as universidades estaduais e federais no site do Ministério da Educação (MEC). Em seguida, foram localizados os websites de todas as instituições via Google, onde foram coletadas as listas de e-mails das coordenações de curso.

O material da pesquisa foi enviado por e-mail às coordenações, com a solicitação de divulgação aos estudantes matriculados. De acordo com o Censo do MEC, o Brasil possui aproximadamente 9 milhões de universitários, sendo que apenas 23,2% (2.079.123) das matrículas estão concentradas em instituições públicas^(20)^. Para determinar o tamanho da amostra, foi considerado um nível de confiança de 95% e uma margem de erro de 3%, resultando em uma amostra mínima de 1.066 estudantes. O cálculo foi realizado por meio de uma calculadora amostral online. A coleta de dados ocorreu entre novembro de 2021 e março de 2022.

Os alunos cursavam cursos das Ciências da Saúde, Ciências Sociais Aplicadas, Linguística, Letras e Artes, Engenharias, Ciências Biológicas e Ciências Agrárias, obtendo representatividade de todas as áreas e de todos os estados brasileiros e unidades federativas.

### Análise dos Dados

A sensibilidade psicométrica dos itens do EAT foi avaliada por medidas de tendência central, dispersão, curtose e assimetria, considerando adequados itens com coeficientes de assimetria < 3 e curtose < 7^(21)^. A consistência interna foi estimada pelo coeficiente alfa de Cronbach (α), considerando α ≥ 0,70 como adequado. A adequação da amostra foi verificada pelo índice de Kaiser-Meyer-Olkin (KMO), sendo considerado adequado um valor superior a 0,5, e pelo teste de esfericidade de Bartlett (p < 0,005).

Para avaliar a correlação entre os fatores, utilizou-se o teste de correlação de Spearman, considerando: 0,8 a 1,0 como forte correlação; 0,5 a 0,8 como correlação moderada; 0,2 a 0,5 como correlação fraca; e 0,0 a 0,2 como correlação insignificante.

Foi realizada análise fatorial confirmatória (AFC) para avaliar a plausibilidade da estrutura multidimensional do EAT. A AFC é uma abordagem que pode ser empregada, entre diversas finalidades, para determinar se a estrutura fatorial de um modelo proposto está alinhada com o conceito teórico do construto em uma amostra específica. Sendo um teste de caráter confirmatório, é necessário que a estrutura fatorial seja definida pelo pesquisador antes da execução do teste^(22)^.

A análise foi implementada utilizando o método de estimação Robust Diagonally Weighted Least Squares (RDWLS), com uma avaliação do modelo configural para verificar a equivalência das categorias dos itens entre homens e mulheres^(23,24)^. Os índices de ajuste utilizados incluíram: qui-quadrado (χ²), razão de qui-quadrado por graus de liberdade (χ²/gl), *Comparative Fit Index* (CFI), *Tucker-Lewis Index* (TLI), *Standardized Root Mean Residual* (SRMR) e *Root Mean Square Error of Approximation* (RMSEA). Valores de χ² não devem ser significativos; a χ²/gl deve ser < 5 ou, preferencialmente, < 3; CFI e TLI devem ser > 0,90, preferencialmente acima de 0,95; RMSEA deve ser < 0,08, preferencialmente < 0,06, com intervalo de confiança (limite superior) < 0,10^(25)^.

Considerar-se-ão cargas fatoriais adequadas acima de 0,40^(26)^. Além da aplicação da escala, foram coletadas e analisadas características sociodemográficas como: idade, sexo, situação conjugal, estado no qual reside, renda familiar e qual a área de sua graduação.

Os resultados foram organizados em planilha do Excel e exportados para o *Statistical Package for the Social Sciences* (SPSS), versão 23.0, para análises descritivas, inferenciais e correlacionais. A AFC foi realizada no *Jeffreys’s Amazing Statistics Program* (JASP).

## RESULTADOS

A amostra foi composta por 5.292 estudantes universitários de todas as regiões do Brasil, com média de idade de 24 anos (DP ± 6,9). Em relação ao sexo, 67,4% (n=3.565) eram mulheres; 45,5% (n=2.408) se autodeclararam da raça branca; 86,5% (n=4.577) eram solteiros; e 49,5% (n=2.619) possuíam renda familiar entre 1 e 5 salários mínimos. Houve representantes de todas as regiões do país, com maior quantitativo de estudantes das Ciências Humanas (20,8%, n=1.101) e de instituição federal (57,9%, n=3.066).

Realizou-se a correlação entre os fatores das escalas. Embora os fatores tenham apresentado correlações significativas (p=0,000), observou-se uma correlação moderada entre o fator Dieta e o fator Bulimia e Preocupação com Alimentos (rô=0,637), enquanto o fator Controle Oral apresentou correlação fraca com os demais fatores (rô=0,284 e rô=0,209). Dados descritivos apontam a sensibilidade psicométrica dos itens do EAT (Tabela 1). Os valores de achatamento (*kurtosis*) não foram significativos, apresentando pequenas variações de -1,456 a 6,780.

**Tabela 1:** Medidas de resumo e de distribuição por itens do Teste de Atitudes Alimentares para estudantes universitários brasileiros (N=5.292), Fortaleza, Ceará, Brasil, 2021–2022

Item	Média	Desvio-Padrão	Assimetria	Curtose
I1) Fico apavorada com a ideia de estra engordando	1,01	1,242	0,662	-1,277
I2) Evito comer quando estou com fome	0,28	0,741	2,685	6,046
I3) Sinto-me preocupada com os alimentos	0,72	1,050	1,172	-0,081
I4) Continuar a comer em exagero faz com que eu sinta que não sou capaz de parar	0,66	1,093	1,310	0,075
I5) Corto meus alimentos em pedaços pequenos	0,48	0,928	1,820	1,922
I6) Presto atenção na quantidade de calorias dos alimentos que eu como	0,35	0,846	2,318	3,965
I7) Evito, particularmente, alimentos com alto teor de carboidratos (pão, batata, arroz)	0,28	0,752	2,705	6,138
I8) Sinto que os outros gostariam que eu comesse mais.	0,54	1,038	1,622	0,972
I9) Vomito depois e comer	0,14	0,569	2,239	6,000
I10) Sinto-me extremamente culpada depois de comer	0,56	1,044	1,561	0,791
I11) Preocupo-me com o desejo de ser mais magra	0,89	1,228	0,877	-0,984
I12) Penso em queimar calorias a mais quando me exercito	0,89	1,209	0,857	-0,977
I13) As pessoas me acham muito magra	0,56	1,036	1,565	0,818
I14) Preocupo-me com a ideia de haver gordura no meu corpo	0,86	1,202	0,907	-0,892
I15) Demoro mais tempo para fazer as refeições do que as outras pessoas	0,69	1,117	1,260	-0,082
I16) Evito alimentos que contenham açúcar	0,34	0,806	2,369	4,378
I17) Costumo comer alimentos dietéticos?	0,27	0,739	2,798	6,652
I18) Sinto que os alimentos controlam minha vida	0,44	0,946	1,940	1,203
I19) Demonstro autocontrole diante dos alimentos	0,87	1,105	0,851	-0,785
I20) Sinto que os outros me pressionam para comer	0,43	0,923	1,997	2,477
I21) Passo muito tempo pensando em comer	0,62	1,033	1,405	0,443
I22) Sinto desconforto após comer doces	0,45	0,948	1,891	2,032
I23) Faço regimes para emagrecer	0,44	0,933	1,940	2,238
I24) Gosto de sentir meu estômago vazio	0,31	0,816	2,566	5,113
I25) Gosto de experimentar novos alimentos ricos em calorias	1,28	1,187	0,268	-1,456
I26) Sinto vontade de vomitar após as refeições	0,24	0,729	3,018	6,780

*N – número de participantes.*

O α de Cronbach do fator Dieta foi α = 0,827; Bulimia, α = 0,770; e Controle Oral, α = 0,725. A consistência interna do EAT apresentou-se adequada (α = 0,889), com índice de KMO de 0,921 e teste de esfericidade de Bartlett com p < 0,001.

Realizou-se AFC objetivando avaliar a plausibilidade da estrutura multidimensional do EAT(28). A estrutura apresentou bons resultados de ajuste. Obteve-se χ² = 14.447,944 (296 gl), p < 0,001. A razão qui-quadrado por graus de liberdade foi elevada (48,81), com CFI = 0,843, TLI = 0,828, SRMR = 0,125 e RMSEA = 0,095 [0,094–0,096], coerente com critérios de ajustes insatisfatórios previamente adotados para a estrutura fatorial nessa amostra. As cargas fatoriais dos itens foram elevadas, variando de 0,43 (item I7) até 1,31 (item I16) (Modelo 01).

O item I10 – Sinto-me extremamente culpada depois de comer (λ = 0,980) apresentou altas cargas cruzadas com I8 – Sinto que os outros gostariam que eu comesse mais (λ = 0,886), I13 – As pessoas me acham muito magra (λ = 0,931) e I20 – Sinto que os outros me pressionam para comer (λ = 0,634).

Objetivando identificar o melhor ajuste, realizaram-se outras estruturas fatoriais para o refinamento do modelo original, retirando os itens que obtiveram menores cargas fatoriais cruzadas. O Modelo 02 foi realizado ao retirar o I20, o Modelo 03 ao retirar os itens I20 e I8, e o Modelo 04 obteve-se ao retirar os itens I20, I8 e I13 (Tabela 2).

**Tabela 2:** Índices de qualidade de ajuste da Análise Fatorial Confirmatória do Teste de Atitudes Alimentares aplicada em universitários, Fortaleza, Ceará, Brasil, 2021–2022

Estimativas	Modelo 01	Modelo 02	Modelo 03	Modelo 04
χ^2^	14447,944	12365,644	9974,759	6748,821
χ^2^/gl	48,81	45,46	40,05	29,73
CFI	0,843	0,858	0,880	0,916
TLI	0,828	0,844	0,867	0,907
RMSEA	0,095 (0,094-0,096)	0,092 (0,090-0,093)	0,086 (0,084-0,087)	0,074 (0,072-0,075)
SRMR	0,125	0,120	0,114	0,097

*CFI - Comparative Fit Index; TLI - Tucker Lewis Index; RMSEA - Root-Mean-Square Error of Aproximation; SRMR - Standardized root mean square residuals.*

Todos os ajustes apresentados no modelo 04 são aceitáveis para o modelo trifatorial original da escala. Realizou-se a Análise Fatorial Confirmatória Multigrupo (AFC-MG) de forma a investigar se há invariância do EAT para homens e mulheres. Realizou-se a análise para o modelo original (modelo 01) e o modelo 04 que apresentou o melhor ajuste para a amostra testada (Tabela 3).

**Tabela 3:** Índices de invariância configural da Análise Fatorial Confirmatória Multigrupo (gênero) do Teste de Atitudes Alimentares aplicada em universitários, Fortaleza, Ceará, Brasil, 2021-2022

Estimativas	Modelo 01	Modelo 04
χ^2^	14443,225	6748,821
χ^2^/gl	24,39	14,82
CFI	0,847	0,920
TLI	0,832	0,920
RMSEA	0,094 (0,093-0,095)	0,0742 (0,071-0,074)
SRMR	0,125	0,097

CFI - Comparative Fit Index; TLI - Tucker Lewis Index; RMSEA - Root-Mean-Square Error of Aproximation; SRMR - Standardized root mean square residuals.

A invariância configural demonstrou que o modelo 04 da EAT configura-se como uma medida equivalente para homens e mulheres, o que permite que, realizado refinamento do instrumento, este permite a comparação entre grupos. Na Figura 1 apresentamos a carga fatorial do instrumento após ajuste e na Figura 2 as cargas fatoriais relacionadas aos grupos (masculino e feminino).

**Figura 1:** Análise fatorial do Teste Atitudes Alimentares ajustado para amostra de universitários brasileiros (n=5262)

**Figura 2:** Análise fatorial do Teste Atitudes Alimentares ajustado para amostra de universitários brasileiros, sexo feminino e masculino (n=5262)

## DISCUSSÃO

Os resultados deste estudo fornecem insights valiosos sobre a validade fatorial do EAT-26 em estudantes universitários brasileiros, contribuindo significativamente para a literatura sobre instrumentos de triagem de TA nesta população específica.

O EAT-26 é amplamente utilizado para identificar comportamentos alimentares de risco, sendo composto por três fatores principais: Dieta, Bulimia e Preocupação com Alimentos, e Autocontrole Oral. O fator Dieta avalia a tendência a evitar alimentos considerados calóricos e a restrição alimentar, refletindo comportamentos associados à busca por magreza e insatisfação corporal. Estudos indicam que altos escores nesse fator estão correlacionados com práticas restritivas e insatisfação com a imagem corporal, especialmente entre adolescentes do sexo feminino^(27)^.

O segundo fator, Bulimia e Preocupação com Alimentos, investiga episódios de compulsão alimentar seguidos de comportamentos compensatórios, além de uma preocupação excessiva com comida. Os escores elevados nesse domínio estão associados a um maior risco de desenvolvimento de TA, como bulimia nervosa, e frequentemente correlacionam-se com altos níveis de ansiedade e impulsividade^(28)^.

Por fim, o fator Autocontrole Oral relaciona-se ao autocontrole percebido sobre a ingestão alimentar e à pressão social para se alimentar de determinada maneira. Indivíduos com baixos escores nesse fator podem apresentar maior vulnerabilidade a episódios de descontrole alimentar, influenciados por contextos sociais e emocionais^(29)^.

A AFC revelou que, após ajustes, o modelo de três fatores do EAT-26 apresentou índices de ajuste aceitáveis para esta amostra, corroborando sua estrutura original. Este achado é diferente dos encontrados em estudos internacionais recentes. Estudo realizado com crianças na Espanha demonstrou que a AFE apoia uma estrutura multidimensional para a escala, mas com seis fatores^(29)^, indicando que a estrutura fatorial do EAT-26 pode variar dependendo do contexto cultural e etário.

A consistência interna do instrumento mostrou-se adequada (α = 0,889), alinhando-se com estudos internacionais recentes. Uma meta-análise^(6)^ relatou uma confiabilidade média de 0,86 para o EAT-26 em diversas populações. A robustez da consistência interna reforça a confiabilidade do instrumento para uso em contextos acadêmicos brasileiros, um aspecto crucial considerando a prevalência crescente de comportamentos de risco para TA em estudantes universitários^(4,30)^.

Um achado particularmente notável foi a invariância configural demonstrada entre homens e mulheres após o refinamento do instrumento. Este resultado é especialmente relevante à luz das diferenças de gênero frequentemente observadas nos TA. Destaca-se a importância de considerar as diferenças de gênero na manifestação e avaliação dos TA, enfatizando a necessidade de instrumentos que sejam sensíveis a essas variações. A invariância configural encontrada no presente estudo sugere que o EAT-26 ajustado pode ser uma medida equivalente para ambos os sexos, permitindo comparações válidas entre grupos^(31)^.

Evidências de diferenças significativas na prevalência e manifestação dos TA entre os gêneros masculino e feminino têm sido apresentadas na literatura. Embora tradicionalmente associados ao público feminino, existe um aumento notável desses transtornos entre homens, sugerindo que fatores socioculturais e biológicos influenciam ambos os gêneros de maneira distinta. Além disso, homens tendem a relatar vergonha por sofrerem de uma condição frequentemente considerada “feminina”, o que pode levar à subnotificação e a atrasos no diagnóstico^(8,32)^.

Fatores socioculturais, como a pressão para manter uma imagem corporal específica, influenciam ambos os gêneros, mas se manifestam de maneiras distintas. Assim, é crucial que os profissionais de saúde mental estejam atentos às manifestações nesses pacientes, considerando as particularidades de gênero no desenvolvimento e na apresentação clínica desses transtornos^(6,33)^.

A prevalência de comportamentos de risco para TA em estudantes universitários ressalta a importância de ferramentas de triagem confiáveis e válidas^(34)^. O EAT-26, com sua estrutura fatorial confirmada neste estudo, pode servir como um instrumento valioso para identificar precocemente estudantes em risco^(35)^.

Para a enfermagem, esses resultados têm implicações significativas. Enfermeiros que atuam em ambientes universitários ou em cuidados primários podem utilizar o EAT-26 com maior confiança como uma ferramenta de triagem para comportamentos de risco relacionados a TA. A validação da estrutura fatorial e a demonstração de invariância entre gêneros permitem uma aplicação mais ampla e equitativa do instrumento. Isso se alinha com as recomendações sobre a importância de intervenções digitais baseadas em evidências para a saúde mental em estudantes universitários, nas quais ferramentas de triagem validadas desempenham um papel crucial^(35)^.

Além disso, a confirmação da validade do EAT-26 nesta população específica pode informar o desenvolvimento de programas de prevenção e intervenção precoce em saúde mental focados nos TA. Enfermeiros podem desempenhar um papel crucial na implementação desses programas, utilizando o EAT-26 como uma ferramenta de avaliação inicial e monitoramento de progresso.

É importante notar que o estudo identificou a necessidade de ajustes no modelo original, com a remoção de alguns itens para melhorar o ajuste. Isso sugere que, embora o EAT-26 seja uma ferramenta valiosa, pode haver espaço para adaptações culturais ou específicas para a população universitária brasileira. Futuros estudos poderiam explorar o desenvolvimento de versões culturalmente adaptadas do instrumento, potencialmente melhorando sua sensibilidade e especificidade no contexto brasileiro.

### Limitações do estudo

No tocante às limitações, aponta-se que o estudo não avaliou a validade concorrente ou preditiva do instrumento, aspecto importante na avaliação abrangente de instrumentos de triagem para TA. A natureza pontual do estudo não permite inferências sobre a estabilidade temporal da estrutura fatorial identificada.

Embora o estudo tenha fornecido evidências robustas sobre a validade fatorial do EAT-26 em estudantes universitários brasileiros, algumas limitações devem ser consideradas. A coleta de dados por meio de questionário online e autodeclarado pode ter introduzido viés de resposta, uma vez que os participantes podem ter subestimado ou superestimado seus comportamentos alimentares devido a fatores como percepção subjetiva ou estigma social. A representatividade da amostra, embora ampla, pode não refletir igualmente todas as regiões do país e cursos universitários, o que pode influenciar a generalização dos achados.

Outro aspecto a ser considerado é a influência de fatores socioculturais e psicológicos, como ansiedade, depressão e insatisfação corporal, que não foram abordados de maneira aprofundada, mas que desempenham um papel significativo no desenvolvimento de TA. Assim, futuros estudos podem beneficiar-se da inclusão de avaliações clínicas complementares, análises longitudinais e investigações sobre o impacto de fatores emocionais e ambientais na manifestação de comportamentos alimentares disfuncionais.

### Contribuições para a Área da Enfermagem

As potencialidades deste estudo para a área da enfermagem consistem na indicação de uma ferramenta validada para triagem de comportamentos de risco para TA em estudantes universitários, permitindo intervenções precoces, bem como sinalizam a necessidade de desenvolvimento de programas de prevenção e educação em saúde mais direcionados e eficazes, baseados em evidências locais.

Ademais, os achados contribuem para a prática baseada em evidências na enfermagem, fornecendo dados sobre a validade de um instrumento amplamente utilizado, bem como indicam a abertura de caminho para pesquisas futuras sobre adaptações culturais de instrumentos de saúde mental na população brasileira.

## CONCLUSÕES

Em conclusão, este estudo fornece evidências robustas da validade fatorial do EAT-26 em estudantes universitários brasileiros, com implicações importantes para a prática de enfermagem em saúde mental e cuidados primários. A AFC indicou que, após ajustes, o modelo do EAT-26 apresentou invariância configural, configurando-se como medida equivalente para homens e mulheres.

## Data Availability

Os dados de pesquisa estão disponíveis em repositório: https://osf.io/wz6u4/overview.
